# 53. Sex and Race Disparities in Premature Mortality among People with HIV: A 21-Year Observational Cohort Study

**DOI:** 10.1093/ofid/ofab466.053

**Published:** 2021-12-04

**Authors:** Rachael Pellegrino, Peter F Rebeiro, Megan Turner, Amber Davidson, Noelle Best, Chandler Shaffernocker, Timothy Sterling, Jessica L Castilho

**Affiliations:** 1 Vanderbilt University Medical Center, Nashville, Tennessee; 2 Meharry Medical College, Nashville, Tennessee

## Abstract

**Background:**

Since the availability of antiretroviral therapy, mortality rates among people with HIV (PWH) have decreased; however, this trend may fail to quantify premature deaths among PWH. We assessed trends and disparities in all-cause and premature mortality by sex, HIV risk factor, and race, among PWH receiving care at the Vanderbilt Comprehensive Care Clinic from January 1998 – December 2018.

**Methods:**

We examined mortality trends across calendar eras using person-time from clinic entry to date of death or December 31, 2018. We compared mortality rates by demographic and clinical factors and calculated adjusted incidence rate ratios (aIRR) and 95% confidence intervals (CI) using multivariable Poisson regression. For individuals who died, years of potential life lost (YPLL) were obtained from the expected years of life remaining by referencing US sex-specific period life tables at age and year of death; age-adjusted YPLL (aYPLL) rates were also calculated. We examined patient factors associated with YPLL using multivariable linear regression.

**Results:**

Among the 6,531 individuals (51% non-Hispanic [NH] White race, 40% NH Black race, 21% female) included, 956 (14.6%) died. Mortality rates dramatically decreased during the study period (Figure). After adjusting for calendar era, age, injection drug use, hepatitis C virus (HCV), year of HIV diagnosis, history of AIDS-defining illness, CD4 cell count, and HIV RNA at clinic entry, only female sex (aIRR=1.32, 95% CI: 1.13–1.55 vs. males) but not NH Black race (aIRR=1.02, 95% CI: 0.88–1.17 vs. NH White race) was associated with increased mortality. In contrast, aYPLL per 1,000-person years was significantly higher for both female and NH Black PWH (Table 1). In adjusted models including CD4 cell count, HIV RNA, HCV, and year of clinic entry, higher YPLL remained associated with NH Black race, female sex regardless of HIV risk factor, and younger age at HIV diagnosis (Table 2).

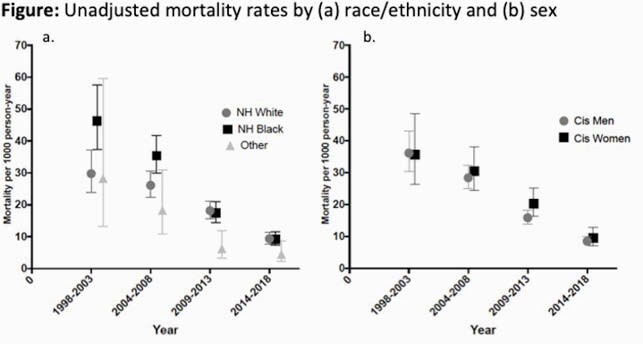

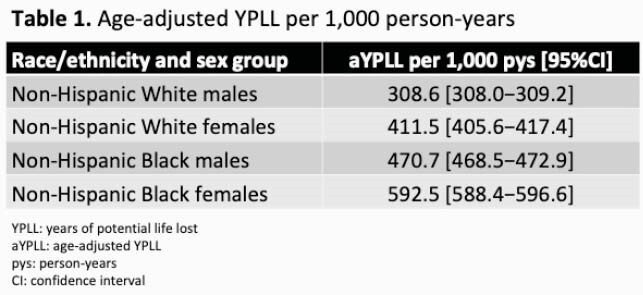

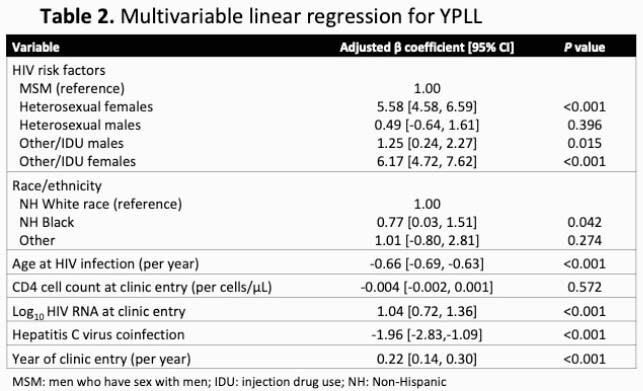

**Conclusion:**

Despite marked improvement over time, sex disparities in mortality as well as sex and race disparities in YPLL remained among PWH in care in this cohort. YPLL is a useful measure for examining persistent gaps in longevity and premature mortality among PWH.

**Disclosures:**

**Peter F. Rebeiro, PhD, MHS**, **Gilead** (Other Financial or Material Support, Single Honorarium for an Expert Panel)

